# Functional validation of *PIK3R1* variant causing activated phosphoinositide 3-kinase-δ syndrome 2 with hypogammaglobulinemia and bronchiectasis

**DOI:** 10.70962/jhi.20250085

**Published:** 2025-08-13

**Authors:** Marat Kribis, Bradly M. Bauman, Cullen M. Dutmer, Jordan Abbott, Jason Catanzaro, Carrie L. Lucas

**Affiliations:** 1 Department of Immunobiology, Yale School of Medicine, New Haven, CT, USA; 2 Department of Medicine, Section of Rheumatology, Allergy and Immunology, Yale School of Medicine, New Haven, CT, USA; 3 Division of Allergy and Immunology, Department of Pediatrics, Emory University School of Medicine, Atlanta, GA, USA; 4 Department of Pediatrics, Section of Allergy and Immunology, University of Colorado School of Medicine, Aurora, CO, USA; 5 Department of Allergy and Immunology, Kaiser Permanente, Denver, CO, USA

## Abstract

A heterozygous *PIK3R1* variant (c.1300-2A>G) was identified in a patient with hypogammaglobulinemia and bronchiectasis. Functional validation revealed aberrant lymphocyte cell states, confirming activated phosphoinositide 3-kinase-δ syndrome 2 and supporting targeted treatment. This work reinforces the importance of precise genetic diagnosis in primary immunodeficiencies.

## Teaser

Authors report a patient with recurrent respiratory infections and bronchiectasis due to an atypical splice-site PIK3R1 variant causing APDS2. Functional studies confirmed PI3Kδ hyperactivation.

## Introduction

Activated phosphoinositide 3-kinase-δ syndrome (APDS) is an inborn error of immunity (IEI) characterized by varying degrees of immunodeficiency, lymphoproliferation, and autoimmunity due to hyperactive signaling of phosphoinositide 3-kinase-δ (PI3Kδ). APDS1 results from heterozygous gain-of-function mutations in *PIK3CD*, encoding the p110 catalytic subunit of PI3Kδ, and APDS2 results from heterozygous mutations in *PIK3R1*, encoding the p85α regulatory subunit of PI3Kδ (1, 2). Higher intrinsic PI3Kδ activity from either of these mutations leads to hyperactivation of the AKT/mTOR pathway and immune dysregulation, characterized by T cell senescence and impaired B cell maturation and function. Clinical manifestations include recurrent lung, ear, and sinus infections, benign and malignant lymphoproliferation, and a broad variety of autoimmune conditions. Additionally, APDS2 has been rarely reported to have features overlapping with short stature, hyperextensibility of joints, hernia, ocular depression, Rieger anomaly, and teething delay (SHORT) syndrome. Genetic diagnosis in APDS is imperative due to the need for lymphoma surveillance, prevention of damage from infections, guidance on vaccinations, and the opportunity for targeted treatment with the PI3Kδ inhibitor leniolisib. Most disease-causing mutations in *PIK3R1* affect the splice donor site of exon 11, resulting in the deletion of amino acids 434–475 within the inter-SH2 domain of the p85α regulatory subunit of PI3Kδ, which regulates the activity of the catalytic p110 subunits (2). Here, we report functional validation of a heterozygous mutation in the *PIK3R1* splice acceptor site of exon 11 (c.1300-2A>G) identified in a patient with recurrent upper respiratory tract infections and bronchiectasis. This report highlights the importance of genetic testing in patients with unexplained immune dysregulation and expands the spectrum of pathogenic mutations in *PIK3R1*.

## Case presentation

A 16-year-old male presented to the Allergy and Immunology Clinic due to recurrent sinusitis, otitis media, and a chronic cough since early childhood. He underwent tonsillectomy and adenoidectomy in early childhood and multiple sinus surgeries in early adolescence; none provided significant clinical improvement. He was treated with courses of oral antibiotics and intranasal corticosteroids and had not required hospitalizations for infections. He had no history of autoimmunity, lymphadenopathy, thrush, atopy, chronic diarrhea, cardiac disease, or joint hypermobility. He was significantly shorter than his fraternal twin, and had facial features mildly reminiscent of SHORT syndrome, including the triangular appearance of his face with a prominent forehead and deep-set eyes, compared with his brother or parents. His weight was in the 10–15th percentile, and previous evaluations by Gastroenterology and Endocrinology for failure to thrive between ages 4 and 7 years were reportedly normal. Family history was negative for immunodeficiency, autoimmunity, or lymphoproliferation.

Complete blood count and chemistry profile were normal. Serum immunoglobulin levels were low, with IgG of 75 mg/dl (reference range, 528-2,190 mg/dl), IgM 41 mg/dl (reference range, 48–266 mg/dl), and IgA <10 mg/dl (reference range, 44–441 mg/dl). He had incomplete immunization coverage but received tetanus/diphtheria/pertussis (Tdap), measles/mumps/rubella (MMR), and Varicella, with subsequent testing showing nonprotective titers against tetanus, diphtheria, and Varicella. Clinical flow cytometry showed inverted CD4/CD8 T cell ratio at 0.71 (reference range, 1.2–6.2); increase in CD4^+^/CD45RO^+^ T cells at 58.1% (reference range, 18–38%); decrease in CD4^+^CD45RA^+^CD31^+^ cells at 23.7% (reference range, 33.4–61.8%); CD19^+^ cells at the lower limit of normal, with absolute number of 154 cells/μl and relative number of 7.8% (reference ranges, 110–570 cells/μl and 6–23%, respectively); and a marked increase in CD38^hi^/IgM^hi^ B cells at 45.9% (reference range, 0.3–9.2%). Mitogen-induced lymphocyte proliferation assays to phytohemagglutinin and pokeweed mitogen were normal.

While awaiting the initiation of intravenous immunoglobulin (IVIG) replacement therapy, he required a brief hospitalization for a respiratory infection with positive PCR testing for SARS-CoV-2, rhinovirus, Bordetella pertussis, and adenovirus. High-resolution computed tomography obtained prior to admission showed diffuse bilateral bronchiectasis with bronchial wall thickening. There was no lymphadenopathy or splenomegaly. He received nirmatrelvir/ritonavir and azithromycin and was started on IVIG prior to discharge (500 mg/kg monthly), which resulted in clinical improvement and reduced frequency of infections.

## Genetic diagnosis and functional validation

**Figure 1.**
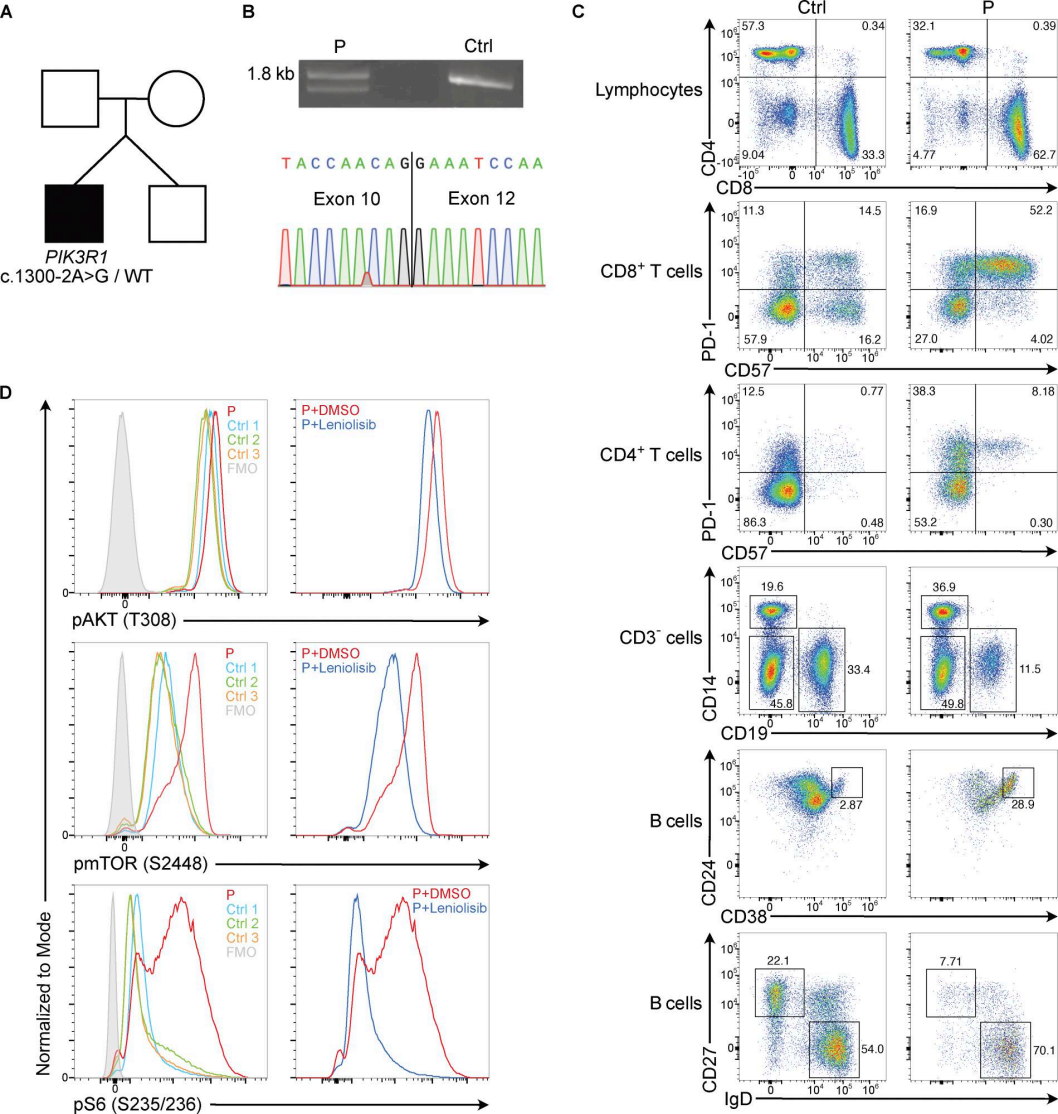
***PIK3R1* c.1300-2A>G variant causes activated PI3Kδ syndrome 2. (A)** Family pedigree with the patient shaded black and the unaffected family members white. **(B)** Amplification and sequencing of *PIK3R1* cDNA between exons 2 and 15; mRNA was extracted from T cell blasts of patient (P) and healthy control subject (Ctrl) 3. PCR products were visualized on an agarose gel (top). Sequencing of the PCR product with Oxford Nanopore Technology showed loss of exon 11 in one of two consensus sequences from P (bottom); the WT sequence is not shown. **(C)** Representative flow cytometric analysis of PBMC from Ctrl 3 and P (prior to leniolisib therapy). **(D)** Flow cytometric analysis of the phosphorylation of AKT at threonine residue 308, mTOR at serine residue 2448, and S6 at serine residues 235/236 in expanded T cell blasts from P and Ctrl 1–Ctrl 3 after treatment with DMSO for 30 min (left), and the same analysis of T cell blasts from P after a 30-min incubation with 500 nM leniolisib vs. DMSO (right). Left and right columns in panel D represent the same experiment. DMSO, dimethyl sulfoxide. Source data are available for this figure: SourceData F1.

Subsequent genetic testing for IEI identified a heterozygous mutation in the exon 11 splice acceptor site (c.1300-2A>G) of *PIK3R1* (Fig. 1 A), classified as likely pathogenic by ClinVar. The mutation was confirmed by Sanger sequencing. Both parents and the patient’s fraternal twin brother tested negative for the mutation. A previous report of the c.1300-2A>G variant in a patient with lymphoproliferation and systemic lupus erythematosus demonstrated abnormal splicing by cDNA analysis, resulting in either complete exon 11 skipping or the usage of a cryptic splice acceptor site with removal of seven nucleotides from exon 11 (3); one additional case with this mutation was included in a recent Italian cohort with APDS (4). However, no functional testing was previously reported. In our case, amplification and sequencing of *PIK3R1* cDNA between exons 2 and 15 from patient-derived T cells demonstrated a mix of the loss of exon 11 (Fig. 1 B) and the wild-type sequence, as expected in a heterozygous patient. Peripheral blood mononuclear cells from the patient, compared with healthy controls (Fig. 1 C), had inverted CD4^+^/CD8^+^ T cell ratio, a higher proportion of CD57^+^ PD-1^+^ CD8^+^ T cells and CD57^+^ PD-1^+^ CD4^+^ T cells, reduced total CD19^+^ cells, marked increase in transitional B cells (CD19^+^CD20^+^CD24^+^CD38^+^) and naïve B cells (CD19^+^CD20^+^CD27^−^IgD^+^), and decrease in class-switched memory B cells (CD19^+^CD20^+^CD27^+^IgD^−^)—characteristic findings of APDS (1). Flow cytometric analysis of phosphorylation events downstream of PI3Kδ in expanded T cell blasts obtained from the patient (prior to initiation of leniolisib) and three healthy control subjects demonstrated an increase in the phosphorylation of AKT, mTOR, and S6 in the patient T cell blasts, which was reduced to normal levels after incubation with the PI3Kδ inhibitor leniolisib (Fig. 1 D), similar to previous reports (5).

The patient began treatment with the PI3Kδ inhibitor leniolisib, which was associated with improved subjective well-being and immunophenotype changes observed by clinical flow cytometry 3.5 mo after therapy onset, including a reduction in transitional B cells, improvement in the CD4/CD8 ratio, and a shift in T cell memory subsets, with increased proportions of central memory (CCR7^+^CD45RA^−^) and reduced proportions of effector memory (CCR7^−^CD45RA^−^) CD4^+^ and CD8^+^ T cells.

## Conclusion

This case expands the molecular spectrum of APDS2 by demonstrating that the atypical *PIK3R1* splice acceptor mutation c.1300-2A>G produces canonical exon 11 skipping and drives constitutive PI3Kδ–AKT/mTOR signaling, resulting in the characteristic immunophenotype of T cell senescence and impaired class switch in B cells. Clinically, the mutation manifested with recurrent respiratory infections and bronchiectasis without overt lymphoproliferation or autoimmunity, highlighting the phenotypic heterogeneity of APDS2 and the risk of underdiagnosis when recurrent hospitalizations, lymphadenopathy, or related features are absent. Features of concomitant SHORT syndrome may be subtle and incomplete. Early next-generation sequencing should be pursued in patients with otherwise unexplained antibody deficiency and/or chronic airway disease.

## Human subjects research

All human subjects in the present study provided informed consent to use their samples for research, in accordance with the Helsinki principles for enrollment in research protocols. Approval was obtained from the Institutional Review Board of Yale University (Human Investigation Committee protocol no. 1605017838).

## Supplementary Material

SourceData F1 is the source file for Fig. 1.
