# Supplementary figures and images for: Functional validation of PIK3R1 variant causing activated phosphoinositide 3-kinase-δ syndrome 2 with hypogammaglobulinemia and bronchiectasis
Source: J Hum Immun. 2025 Aug 13;1(3):e20250085. doi: 10.70962/jhi.20250085 (PMC13177672; doi:10.70962/jhi.20250085)

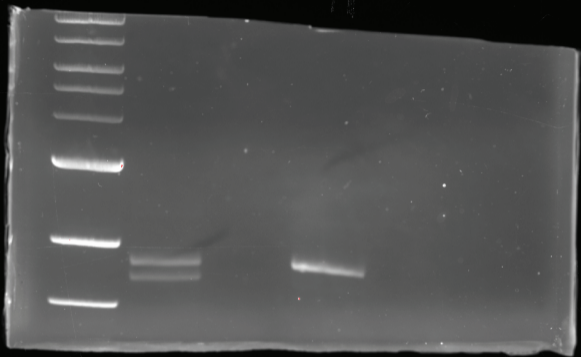

Supplement: SourceData F1 — is the source file for Fig. 1. [file jhi_20250085_sourcedataf1.pdf]
